# Optimal hyperthermic intraperitoneal chemotherapy regimen for advanced and peritoneal metastatic gastric cancer: a systematic review and Bayesian network meta-analysis

**DOI:** 10.3389/fonc.2024.1466473

**Published:** 2024-11-12

**Authors:** Tianqi Wang, Shengjie Ma, Shiwei Zhang, Yilihaer Aizezi, Quan Wang

**Affiliations:** Department of Gastric and Colorectal Surgery, General Surgery Center, The First Hospital of Jilin University, Changchun, China

**Keywords:** gastric cancer, HIPEC, chemotherapy, systematic review, Bayesian network meta-analysis

## Abstract

**Background:**

Peritoneal metastasis is one of the most common modes of spread of gastric cancer. Currently, surgical treatment combined with hyperthermic intraperitoneal chemotherapy (HIPEC) and systemic chemotherapy has demonstrated promising outcomes in both the treatment and prevention of peritoneal metastasis in gastric cancer. However, various HIPEC drug regimens are in clinical use, and their efficacy remains unclear. This study aims to evaluate the effectiveness of different HIPEC drug regimens in patients with advanced gastric cancer to determine the optimal therapeutic approach.

**Methods:**

This study conducted a systematic review and Bayesian network meta-analysis. Patients in the experimental group underwent surgery combined with HIPEC and chemotherapy. The search period covered literature from database inception to June 1, 2024. Hazard ratios (HRs) with 95% confidence intervals (CIs) were used to evaluate overall survival (OS) as the primary outcome. Odds ratios (ORs) with 95% CIs were used to assess overall disease recurrence, peritoneal recurrence, and postoperative morbidity as secondary outcomes. To ensure scientific rigor and transparency, this study has been registered with PROSPERO (CRD42024533948).

**Results:**

A total of 11 randomized controlled trials (RCTs) involving 1092 patients were included. Compared to surgery combined with chemotherapy, the regimens of cisplatin (HRs = 0.52, 95% CI: 0.38-0.73), mitomycin C (HRs = 0.99, 95% CI: 0.55-1.79), cisplatin plus fluorouracil (HRs = 0.60, 95% CI: 0.38-0.95), and oxaliplatin plus 5-fluorouracil (HRs = 0.53, 95% CI: 0.36-0.78) all demonstrated benefits in OS. The cisplatin (ORs = 0.16, 95% CI: 0.03-0.60) and mitomycin C (ORs = 0.03, 95% CI: 0-0.71) regimens also showed advantages in reducing peritoneal recurrence, with no impact on postoperative morbidity. Importantly, the cisplatin regimen was superior to other regimens in terms of OS and overall disease recurrence, achieving a balance between efficacy and safety.

**Conclusions:**

Compared to chemotherapy alone, HIPEC treatment shows significant benefits in OS without a notable disadvantage in postoperative morbidity. Although no single HIPEC regimen demonstrated clear benefits across all outcomes, the cisplatin regimen performed well in multiple aspects, indicating its potential for further research and clinical application.

**Systematic review registration:**

https://www.crd.york.ac.uk/PROSPERO/display_record.php?RecordID=533948, identifier CRD42024533948.

## 1 Introduction

Gastric cancer (GC) ranks as the fifth most prevalent malignancy and the second leading cause of cancer-related deaths globally (1). Despite recent declines in both incidence and mortality rates, over one million new cases are still diagnosed annually worldwide (2). A significant concern is the predominance of advanced-stage GC in both China and Western countries, with 10% to 35% of cases considered unresectable (3, 4). Moreover, 14% of GC patients present with peritoneal metastasis at diagnosis, leading to a median survival of 4-12 months and a 5-year survival rate of less than 5% (5, 6). Research indicates that patients with advanced GC who do not undergo chemotherapy or surgery have a median survival of just 7 months and a 1-year survival rate of 22.2% (7). Reliance solely on surgical resection or systemic chemotherapy has demonstrated limited efficacy in improving survival rates; even with radical tumor resection and lymphadenectomy, early recurrence and tumor progression are common. Studies show that the peritoneum is the most frequent site of recurrence following GC treatment, with 15% of advanced GC patients having synchronous peritoneal metastases and approximately 35% dying from peritoneal metastases post-surgery (8). Additionally, due to the plasma-peritoneal barrier, the intraperitoneal concentration of intravenously administered chemotherapeutic agents remains exceedingly low, rendering palliative chemotherapy insufficient to significantly improve patient outcomes (9, 10).

Hyperthermic intraperitoneal chemotherapy (HIPEC) has been actively utilized in the treatment of gastric cancer with peritoneal metastasis. Initially introduced by Spratt et al. (11) in 1980 for patients with pseudomyxoma peritonei,HIPEC eliminates tumor cells through several mechanisms. Firstly, the continuous flow of perfusion fluid exerts a mechanical flushing effect on peritoneal implants and free-floating cancer cells in the abdominal cavity. Additionally, the high concentration of anti-cancer drugs within the abdominal cavity directly eradicates free cancer cells and residual microscopic disease. During treatment, normal tissue cells can withstand temperatures of 47°C for 1 hour, whereas tumor cells begin to die at 43°C within the same duration (12). HIPEC maintains the intraperitoneal temperature at (43.0 ± 0.2)°C for over an hour, causing irreversible damage to cancer cells while minimizing harm to normal cells (13). Moreover, the elevated temperature increases the permeability of cancer cell membranes and tumor vasculature, enhancing the penetration and absorption of chemotherapeutic agents (14). Under high temperatures, the penetration depth of chemotherapeutic drugs can increase from approximately 1 mm to 5 mm, significantly augmenting the synergistic effects of hyperthermia and chemotherapy (15–17). These combined effects contribute to the efficacy of HIPEC in treating tumors with peritoneal metastasis.

Currently, the integration of surgical treatment and hyperthermic intraperitoneal chemotherapy (HIPEC) is gaining increasing clinical application. HIPEC can be administered postoperatively to target the abdominal cavity, thereby preventing the peritoneal spread of gastric cancer. Numerous clinical studies have demonstrated that the combination of HIPEC and surgery not only provides significant survival benefits for patients with peritoneal metastasis of gastric cancer but also shows marked advantages in terms of recurrence-free survival and overall recurrence rates (18, 19). In recent years, for advanced gastric cancer, the combined approach of surgery, HIPEC, and chemotherapy has been increasingly adopted in clinical practice. Neoadjuvant chemotherapy can improve the R0 resection rate, thereby enhancing patient prognosis, while HIPEC specifically prevents and treats the occurrence and progression of peritoneal metastasis. The integration of these treatments with surgery can significantly improve patient outcomes and disease-free survival rates (20).

However, for patients with advanced gastric cancer, clinical studies combining surgery, HIPEC, and chemotherapy remain limited. Although evidence suggests benefits, there is considerable variability in the HIPEC drugs used across different studies, as well as in the application protocols, resulting in a lack of overall consensus. Furthermore, the safety of HIPEC is a significant concern due to the high temperatures and direct stimulation by chemotherapeutic drugs in the abdominal cavity. The combined toxicity of these treatments and their impact on patients are not yet fully understood.

This study aims to explore the selection and advantages of HIPEC regimens for patients with advanced gastric cancer, using survival outcomes and complications as endpoints, based on the currently available randomized controlled trials.

## 2 Materials and methods

This network meta-analysis (NMA) adheres to the Preferred Reporting Items for Systematic Reviews and Meta-Analysis (PRISMA) extension statement for network meta-analyses (**Supplementary Table 1**). Given the lack of randomized controlled trials directly comparing different HIPEC combined chemotherapy and surgical regimens, we employed a Bayesian approach for indirect comparisons, enabling probabilistic predictions of treatment efficacy and safety. To ensure transparency, reliability, and novelty, this study protocol has been registered with the Prospective Register of Systematic Reviews (CRD42024533948).

### 2.1 Data sources and search strategy

This study systematically searched the PubMed, EMBASE, Cochrane Library, and ClinicalTrials.gov databases. The keywords for the literature search included “Stomach Neoplasm,” “Stomach Cancer,” “Stomach adenocarcinoma,” “randomized clinical trial,” “Hyperthermic Intraperitoneal Chemotherapy,” and “HIPEC” (**Supplementary Table 2**). The search period spanned from the inception of the databases to June 1, 2024, utilizing a combination of free-text and MeSH (Medical Subject Headings) terms.

### 2.2 Selection criteria

This meta-analysis aims to determine the effectiveness of surgery combined with HIPEC and chemotherapy for the prevention and treatment of advanced gastric cancer, with or without peritoneal metastasis. The study is structured according to the PICOS framework, as detailed below:

Inclusion Criteria:

1. Population: Adult patients with histologically confirmed advanced gastric adenocarcinoma, with or without peritoneal metastasis.
2. Intervention: Surgery (primary tumor resection/cytoreductive surgery) combined with HIPEC and chemotherapy (perioperative chemotherapy/chemotherapy).
3. Comparison: Surgery (primary tumor resection/cytoreductive surgery) combined with chemotherapy (perioperative chemotherapy/chemotherapy).
4. Outcomes: Primary outcome: Overall Survival (OS). Secondary outcomes: overall disease recurrence, peritoneal recurrence, and postoperative morbidity.
5. Study Design: Randomized controlled trials (RCTs).

Exclusion Criteria:

1. All non-RCT studies and non-English studies.
2. Studies involving surgery combined with HIPEC and chemotherapy for primary tumors other than gastric cancer.
3. Studies where the full text is not available.
4. Randomized controlled trials with unclear outcome measures.

Before inclusion, studies were screened based on their titles and abstracts. All included randomized controlled trials underwent a double-check by two reviewers to ensure that the data included were up-to-date.

### 2.3 Data extraction and quality assessment

Three researchers independently extracted data from the randomized controlled trials in accordance with the Preferred Reporting Items for Systematic Reviews and Meta-Analyses (PRISMA) guidelines. Any discrepancies were resolved through discussion with a fourth author. The extracted data included trial name, trial design, randomization ratio, source and year of publication, tumor stage, sample size, and the treatment regimens for both experimental and control groups. Outcome measures extracted from each article included the hazard ratios (HRs) for OS and the odds ratios (ORs) for overall disease recurrence, peritoneal recurrence, and postoperative morbidity, along with the corresponding 95% confidence intervals (95% CI).

The assessment of the quality of the included randomized controlled trials (RCTs) was conducted utilizing the Cochrane Risk of Bias Tool (2.0). This tool evaluates five key domains: bias originating from the randomization process, bias due to deviations from the intended interventions, bias resulting from incomplete outcome data, bias in outcome measurement, and bias in the selection of reported results. Each RCT was then classified into three categories: low risk, high risk, or “some concerns,” based on the identified risks.

### 2.4 Statistical analysis

The primary outcome was OS, while the secondary outcomes were overall disease recurrence, peritoneal recurrence, and postoperative morbidity. The effect size for OS was measured using HRs with 95% confidence intervals (95% CI). The effect sizes for overall disease recurrence, peritoneal recurrence, and postoperative morbidity were measured using ORs with 95% confidence intervals (95% CI).

A network meta-analysis was conducted within a Bayesian framework using the “rjags” and “gemtc” packages in R to evaluate the efficacy and safety of frontline immunotherapy combinations in advanced gastric cancer. A fixed-effect model was employed, and three independent Markov chains were established. Each chain underwent 20,000 burn-in iterations and 50,000 sampling iterations. The HRs and ORs derived from the Markov chain iterations were used as effect measures to rank the efficacy and safety of different treatment regimens. The results were visualized through graphical representations to facilitate comparison.

### 2.5 Sensitivity analysis

To ensure optimal alignment with our analysis, we conducted model comparison using the Deviance Information Criterion (DIC). This criterion evaluates the relative goodness of fit for both fixed-effects and random-effects models, with lower DIC values indicating a superior model fit. Consistency between the fixed-effects and random-effects models is confirmed if the DIC difference is less than 5. This method facilitated the selection of the most appropriate model for each analysis cohort, thereby enhancing the precision of our approach.

## 3 Results

### 3.1 Systematic review and characteristics of the included studies

**Figure 1:**
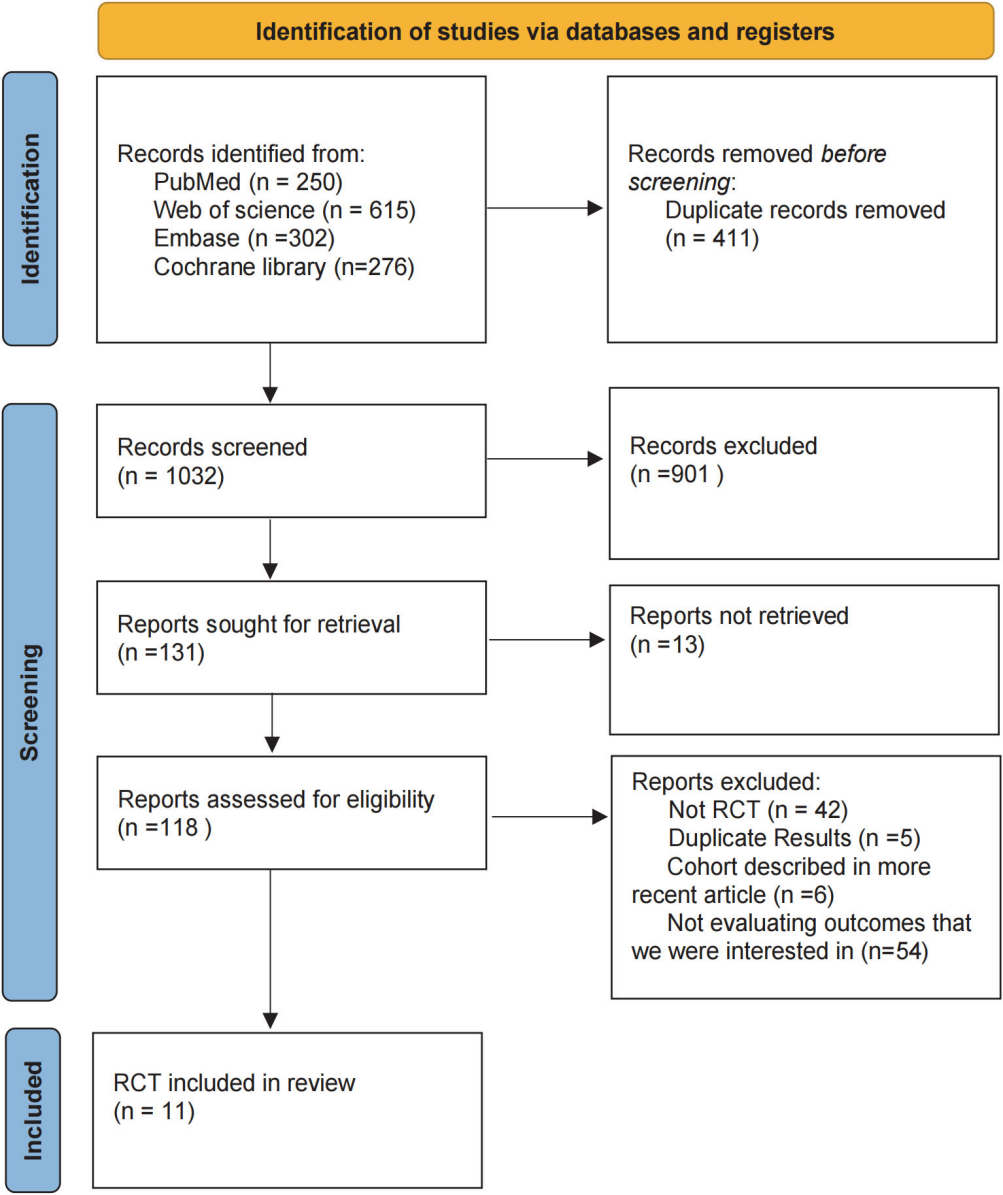
Flowchart of literature retrieval.

In the initial literature search, we retrieved a total of 1,443 records from the databases. After removing duplicates and screening abstracts for relevance, 1,032 studies were deemed eligible for full-text review. Ultimately, 11 studies met our inclusion criteria (**Figure 1**), enrolling a total of 1,092 patients who received one of the following five HIPEC regimens: cisplatin, mitomycin C, cisplatin plus fluorouracil, cisplatin plus mitomycin C, and oxaliplatin plus fluorouracil. Detailed information on all included studies is presented in **Table 1** (19, 21–30). **Table 1** summarizes the 11 included randomized controlled trials (RCTs) that passed the review process. The sample sizes of individual studies ranged from 42 to 192 participants, and the publication years spanned from 1999 to 2023. The research teams were from three different countries, with the majority based in China (n=8), followed by Japan (n=2), and Germany (n=1). Among these studies, two included patients with gastric cancer and peritoneal metastasis, while the remaining studies included patients with advanced gastric cancer without peritoneal metastasis.

**Table 1:** Detailed characteristics of included studies.

Author	Country	Year of publication	Study period	Indication	Selection Criteria	HIPEC Group (n)	Control Group (n)	Control regimes	HIPEC characteristics		
									HIPEC regimes	Duration (min)	Temperature (°C)
Beeharry	China	2019	2014-2015	Prophylaxis	cT3-cT4	40	40	Surg-Chemo	Surg-Chemo-CDDP	60	41-43
Cui	China	2014	2006-2010	Prophylaxis	cT4	96	96	Surg-Chemo	Surg-Chemo-CDDP-FU	90	41-43
Fan	China	2021	2015-2016	Prophylaxis	cT3-cT4	33	17	Surg-Chemo	Surg-Chemo-CDDP	30	42.5-43
Fujimoto	Japan	1999	1987-1996	Prophylaxis	cT4	71	70	Surg-Chemo	Surg-Chemo-MMC	120	43-45
Huang	China	2015	2006-2010	Prophylaxis	cT3-cT4	21	21	Surg-Chemo	Surg-Chemo-CDDP	60	43-45
Liu L	China	2022	2014-2018	Prophylaxis	cT3-cT4	57	57	Surg-Chemo	Surg-Chemo-CDDP	60	40-43
Liu X	China	2020	2010-2012	Prophylaxis	cT3-cT4	64	64	Surg-Chemo	Surg-Chemo-OHP- 5FU	90	41-43
Rau	Germany	2023	2014-2018	Treatment	GCPC	52	53	Surg-Chemo	Surg-Chemo-CDDP-MMC	60	42
Xie	China	2020	2014-2017	Prophylaxis	cT4	51	62	Surg-Chemo	Surg-Chemo-CDDP	60	42-43
Yang	China	2011	2007-2010	Treatment	GCPC	34	34	Surg-Chemo	Surg-Chemo-CDDP-MMC	60-90	42.5-43.5
Kuramoto	Japan	2009	1995-2005	Prophylaxis	cT4	30	29	Surg-Chemo	Surg-Chemo-CDDP	60	-

Surg, Surgery; Chemo, Chemotherapy; CDDP, Cisplatin; MMC, Mitomycin C; FU, Fluorouracil; 5FU, 5 Fluorouracil; OHP, Oxaliplati.

The risk of bias assessment is illustrated in **Supplementary Figure 1**. All included studies reported random allocation. Eight studies detailed allocation concealment and were considered to have a low risk of bias, while three studies did not mention allocation concealment and were categorized as having an unclear risk of bias. Regarding the effect of intervention assignment, none of the studies deviated from the intended interventions, resulting in a low risk of bias. In terms of bias due to missing outcome data, ten studies reported no missing data and were considered to have a low risk of bias, while one study did not report on missing data and was rated as having an unclear risk of bias. For bias in outcome measurement, all studies used appropriate measurement methods with no intergroup differences, leading to a low risk of bias classification. Concerning reporting bias, this was judged based on whether all expected outcomes were reported. Ten studies were considered to have a low risk of bias, while one study was rated as having an unclear risk of bias. Overall, seven studies were classified as having a low risk of bias, while four studies were considered to have an unclear risk of bias.

### 3.2 Network meta-analyses

#### 3.2.1 Network diagram

**Figure 2:**
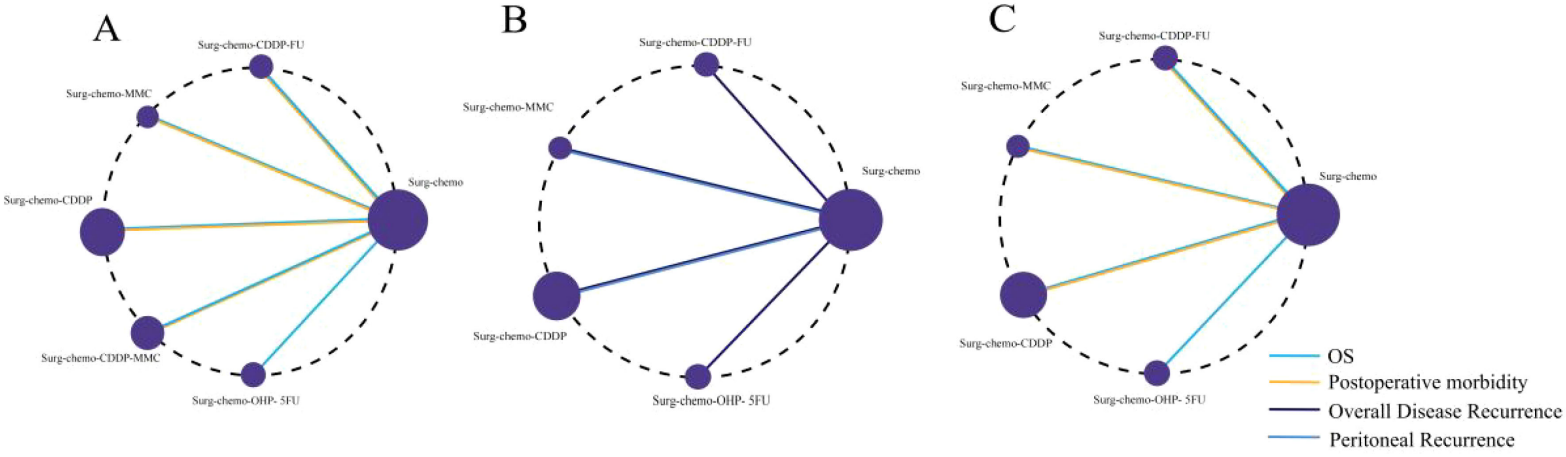
Network of HIPEC regimes with different endpoints. The size of the nodes and the thickness of the edges are weighted according to the number of studies evaluating each treatment and direct comparison, respectively. **(A)** Network of HIPEC regimes with OS and Postoperative morbidity. **(B)** Network of HIPEC regimes with overall disease recurrence and peritoneal recurrence. **(C)** Network of HIPEC regimes of prophylactic studies with Overall survival and Postoperative morbidity.

For all four study endpoints, the network diagrams form closed loops (**Figure 2**). **Figure 2A** illustrates the network diagrams for OS and postoperative morbidity under different HIPEC regimens. The OS diagram includes five HIPEC regimens, while the postoperative morbidity diagram involves four HIPEC regimens. In the OS network, five studies utilized the cisplatin regimen, two studies employed the cisplatin plus mitomycin C regimen, and the mitomycin C, cisplatin plus fluorouracil, and oxaliplatin plus fluorouracil regimens were each represented by one study. In the postoperative morbidity network, six studies used the cisplatin regimen, two studies employed the cisplatin plus mitomycin C regimen, and the mitomycin C and cisplatin plus fluorouracil regimens were each represented by one study.

**Figure 2B** presents the network diagrams for overall disease recurrence and peritoneal recurrence under different HIPEC regimens. The diagram for overall disease recurrence includes four HIPEC regimens, while the peritoneal recurrence diagram includes two HIPEC regimens. For overall disease recurrence, five studies utilized the cisplatin regimen, and one study each employed the mitomycin C, cisplatin plus fluorouracil, and oxaliplatin plus fluorouracil regimens. For peritoneal recurrence, four studies used the cisplatin regimen, and one study utilized the mitomycin C regimen.

We conducted a subgroup analysis of the nine prophylactic studies, and the network diagrams revealed closed loops in both OS and postoperative morbidity endpoints (**Figure 2C**). The OS endpoint included four HIPEC regimens, while the postoperative morbidity endpoint encompassed three. For OS, five studies utilized cisplatin-based regimens, with mitomycin C, cisplatin plus fluorouracil, and oxaliplatin plus fluorouracil each evaluated in one study. In terms of postoperative morbidity, six studies applied the cisplatin regimen, and both the mitomycin C and cisplatin plus fluorouracil regimens were assessed in one study each.

#### 3.2.2 League table

**Figure 3:**
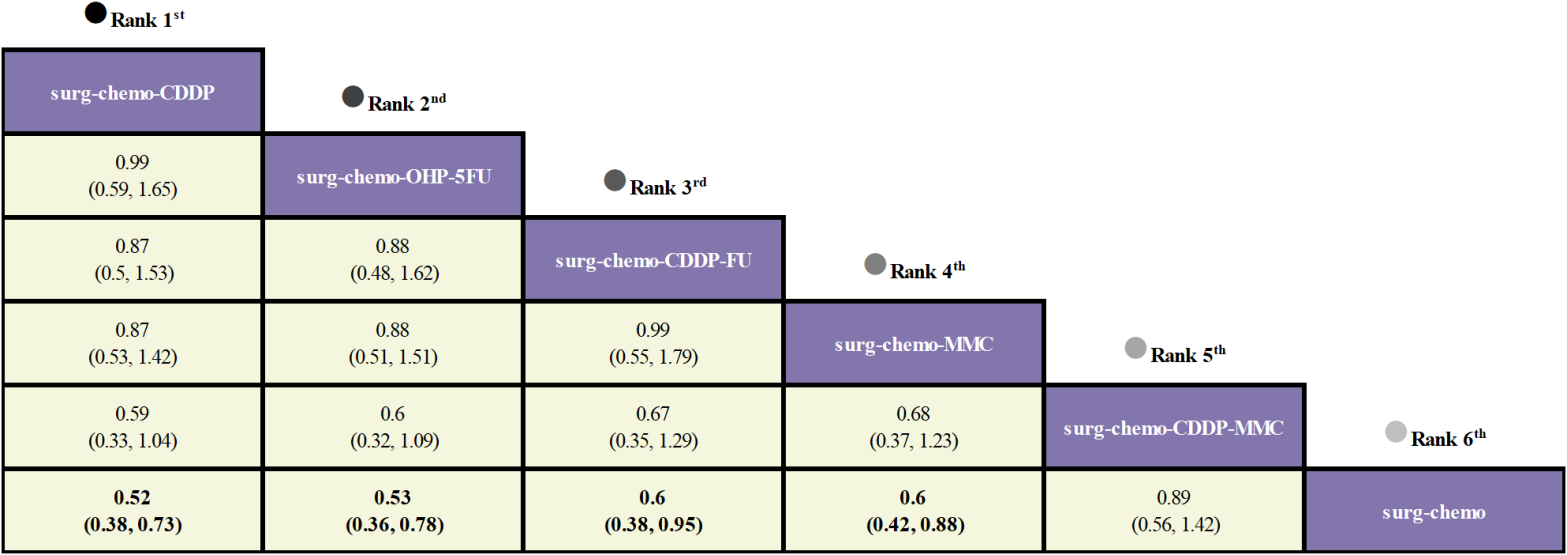
Comparative OS of HIPEC regimens in network meta-analysis. HRs (95% CI) for OS is in cells in common between column-defining and row-defining treatment. Bold cells are significant. For OS, HRs < 1 favors column-defining treatment.

**Figure 4:**
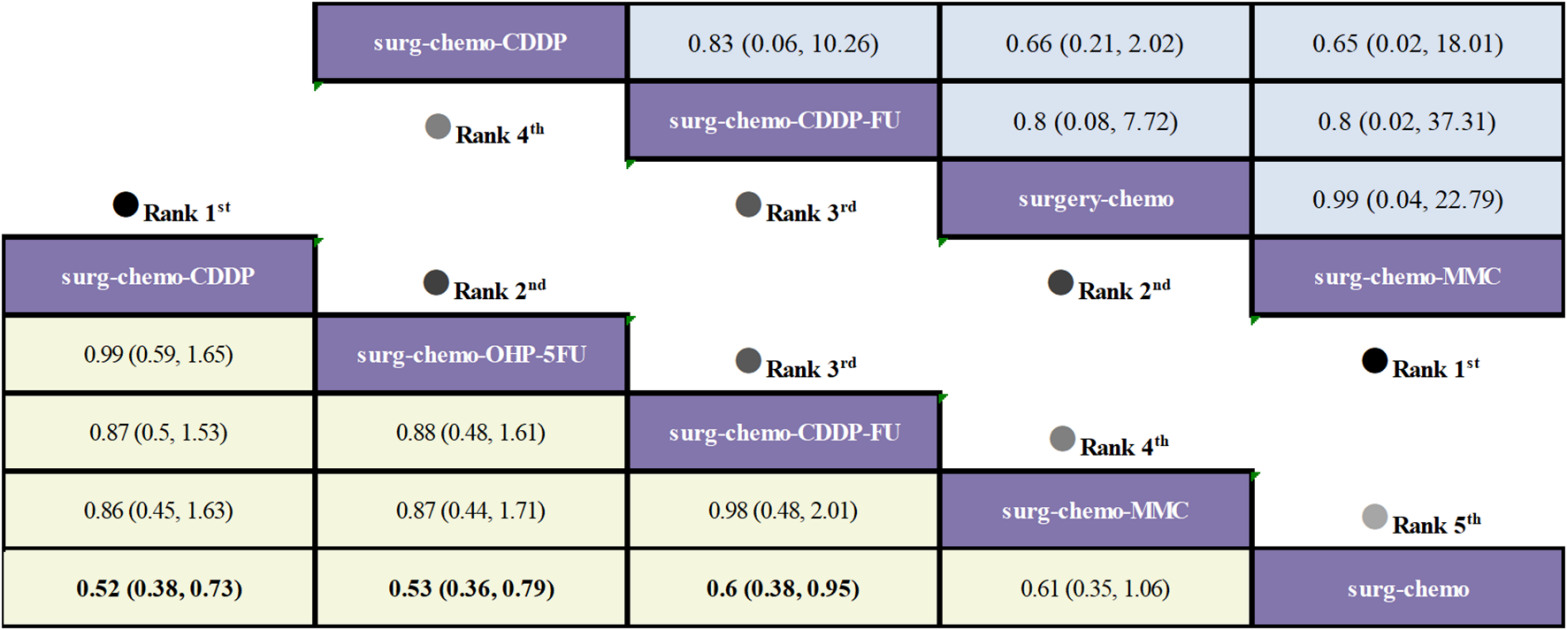
Comparative OS and Postoperative morbidity of HIPEC regimens in network meta-analysis of prophylactic studies. HRs (95% CI) for OS is in cells in common between column-defining and row-defining treatment. ORs (95% CI) for Postoperative morbidity is in cells in common between column-defining and row-defining treatment. Bold cells are significant. For OS, HRs < 1 favors column-defining treatment. For Postoperative morbidity, ORs < 1 favors column-defining treatment.

Regarding OS (**Figure 3**), 10 out of the 11 studies reported OS outcomes post-treatment, encompassing both therapeutic and prophylactic studies, involving five different HIPEC regimens and standard chemotherapy. Compared to chemotherapy alone, the cisplatin regimen (HRs = 0.52, 95% CI: 0.38-0.73), oxaliplatin plus 5-fluorouracil regimen (HRs = 0.53, 95% CI: 0.36-0.78), cisplatin plus fluorouracil regimen (HRs = 0.60, 95% CI: 0.38-0.95), and mitomycin C regimen (HRs = 0.60, 95% CI: 0.42-0.88) all demonstrated significant survival benefits. However, the cisplatin plus mitomycin C regimen (HRs = 0.89, 95% CI: 0.56-1.42) did not show a clear survival advantage. The study also found that the survival benefits among different HIPEC regimens were quite similar. Specifically, the cisplatin regimen and the oxaliplatin plus 5-fluorouracil regimen had comparable benefits (HRs = 0.99, 95% CI: 0.59-1.65), as did the cisplatin plus fluorouracil regimen and the mitomycin C regimen (HRs = 0.99, 95% CI: 0.55-1.79). In the subgroup analysis of prophylactic studies (**Figure 4**), all regimens except cisplatin plus mitomycin C were included. Compared to chemotherapy alone, the cisplatin regimen (HRs = 0.52, 95% CI: 0.38–0.73), oxaliplatin plus 5-fluorouracil regimen (HRs = 0.53, 95% CI: 0.36–0.79), and cisplatin plus fluorouracil regimen (HRs = 0.60, 95% CI: 0.38–0.95) demonstrated a more pronounced survival benefit. In contrast, the mitomycin C regimen (HRs = 0.61, 95% CI: 0.35–1.06) did not show a significant survival advantage. Consistent with the overall analysis, the survival benefit across different HIPEC regimens for OS was quite similar.

**Figure 5:**
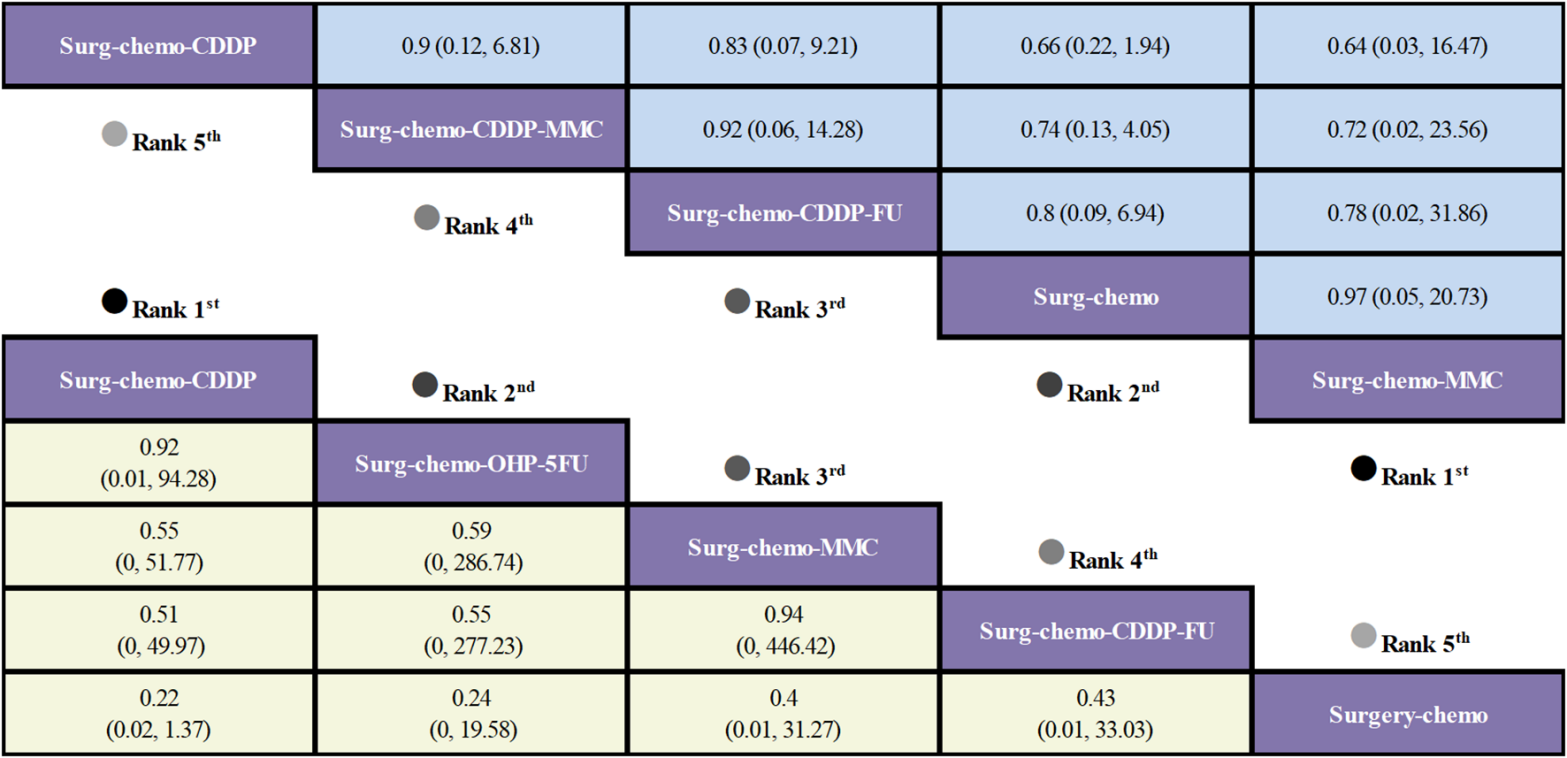
Comparative overall disease recurrence and Postoperative morbidity of HIPEC regimens in network meta-analysis. ORs (95% CI) for overall disease recurrence and Postoperative morbidity is in cells in common between column-defining and row-defining treatment. Bold cells are significant. For overall disease recurrence and Postoperative morbidity, ORs < 1 favors column-defining treatment.

Regarding overall disease recurrence (**Figure 5**), eight of the eleven studies reported outcome measures, and all eight studies were prophylactic. The results indicated that the cisplatin regimen was comparable to the oxaliplatin plus 5-fluorouracil regimen (ORs = 0.92, 95% CI: 0.01-94.28), and the mitomycin C regimen was similar to the cisplatin plus fluorouracil regimen (ORs = 0.94, 95% CI: 0-446.42). Compared to chemotherapy alone, none of the HIPEC regimens demonstrated a significant benefit, nor was there any statistically significant difference among the HIPEC regimens.

**Figure 6:**
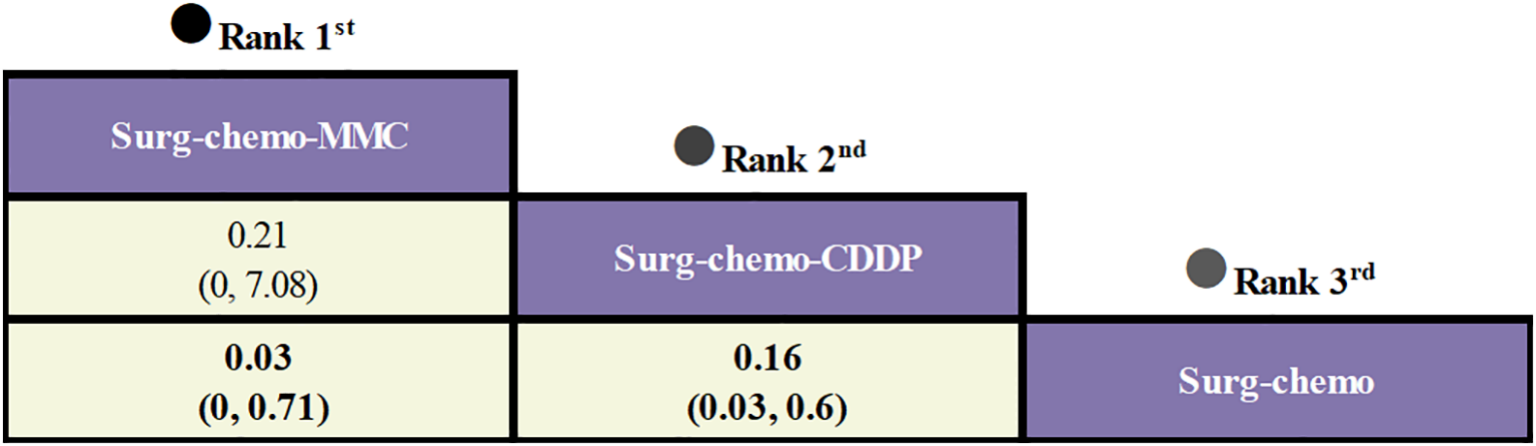
Comparative peritoneal recurrence of HIPEC regimens in network meta-analysis. ORs for peritoneal recurrence is in cells in common between column-defining and row-defining treatment. Bold cells are significant. For peritoneal recurrence, ORs < 1 favors column-defining treatment.

Regarding peritoneal recurrence (**Figure 6**), only five of the eleven studies were included, and all five studies were prophylactic. The results showed that, compared to chemotherapy alone, both the mitomycin C regimen (ORs = 0.03, 95% CI: 0-0.71) and the cisplatin regimen (ORs = 0.16, 95% CI: 0.03-0.60) significantly reduced peritoneal recurrence. Furthermore, there was no significant difference between the two regimens (ORs = 0.21, 95% CI: 0-7.08).

Regarding postoperative morbidity (**Figure 5**), ten of the eleven studies were included, encompassing both therapeutic and prophylactic studies. The results indicated no significant statistical difference in outcomes between various HIPEC regimens and chemotherapy alone. Specifically, the mitomycin C regimen had a comparable postoperative morbidity to chemotherapy (ORs = 0.97, 95% CI: 0.05-20.73). In the subgroup analysis of prophylactic studies (**Figure 4**), no statistically significant differences were observed in outcomes between the various HIPEC regimens and chemotherapy alone. This is consistent with the overall analysis, where postoperative morbidity for the mitomycin C regimen compared to chemotherapy was nearly identical (ORs = 0.99, 95% CI: 0.04–22.79).

#### 3.2.3 Rankings

**Figure 7:**
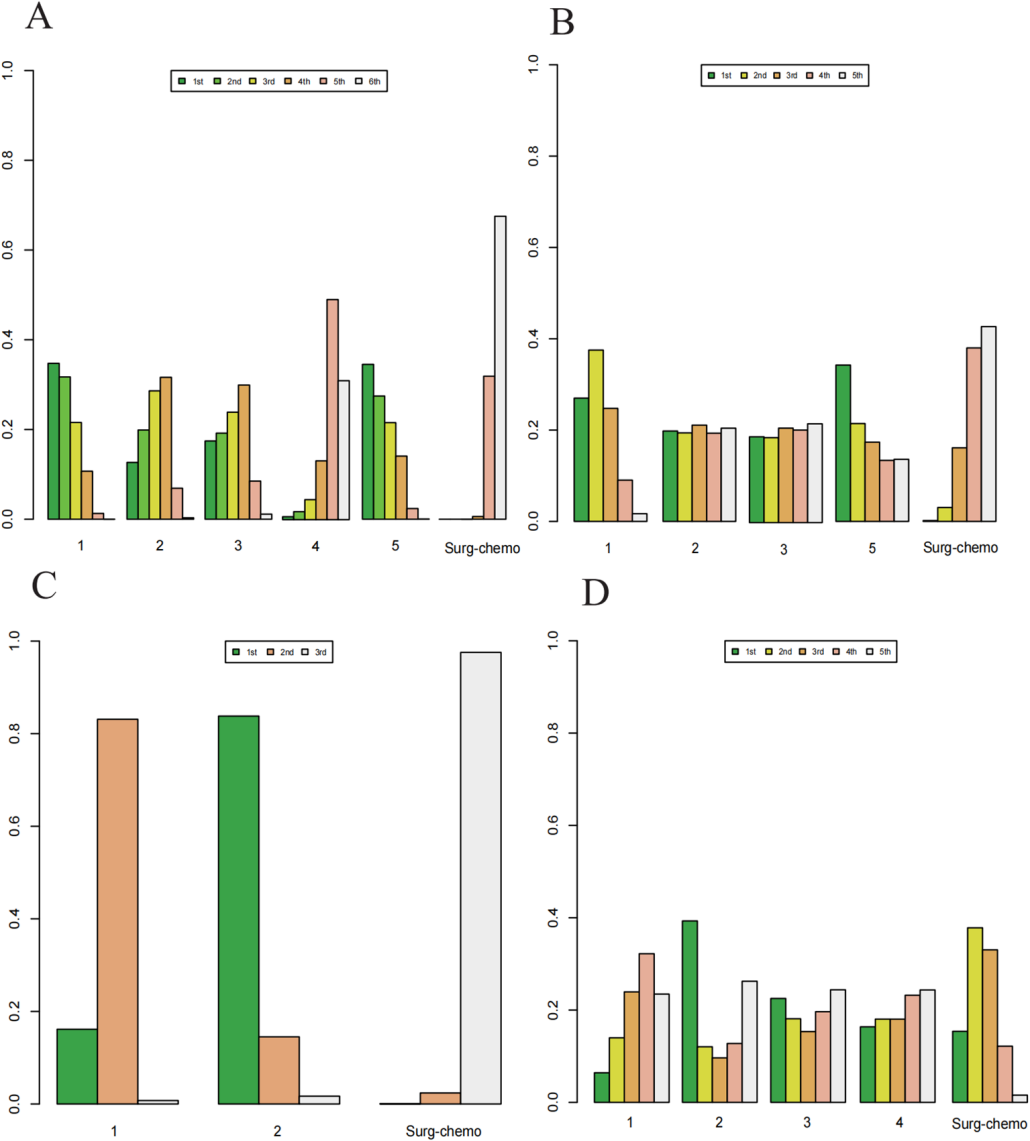
Histogram of possible ranking probabilities for each HIPEC regimen. **(A)** Ranking probability of each HIPEC regimen in OS. **(B)** Ranking probability of each HIPEC regimen in overall disease recurrence. **(C)** Ranking probability of each HIPEC regimen in peritoneal recurrence. **(D)** Ranking probability of each HIPEC regimen in postoperative morbidity.

**Figure 8:**
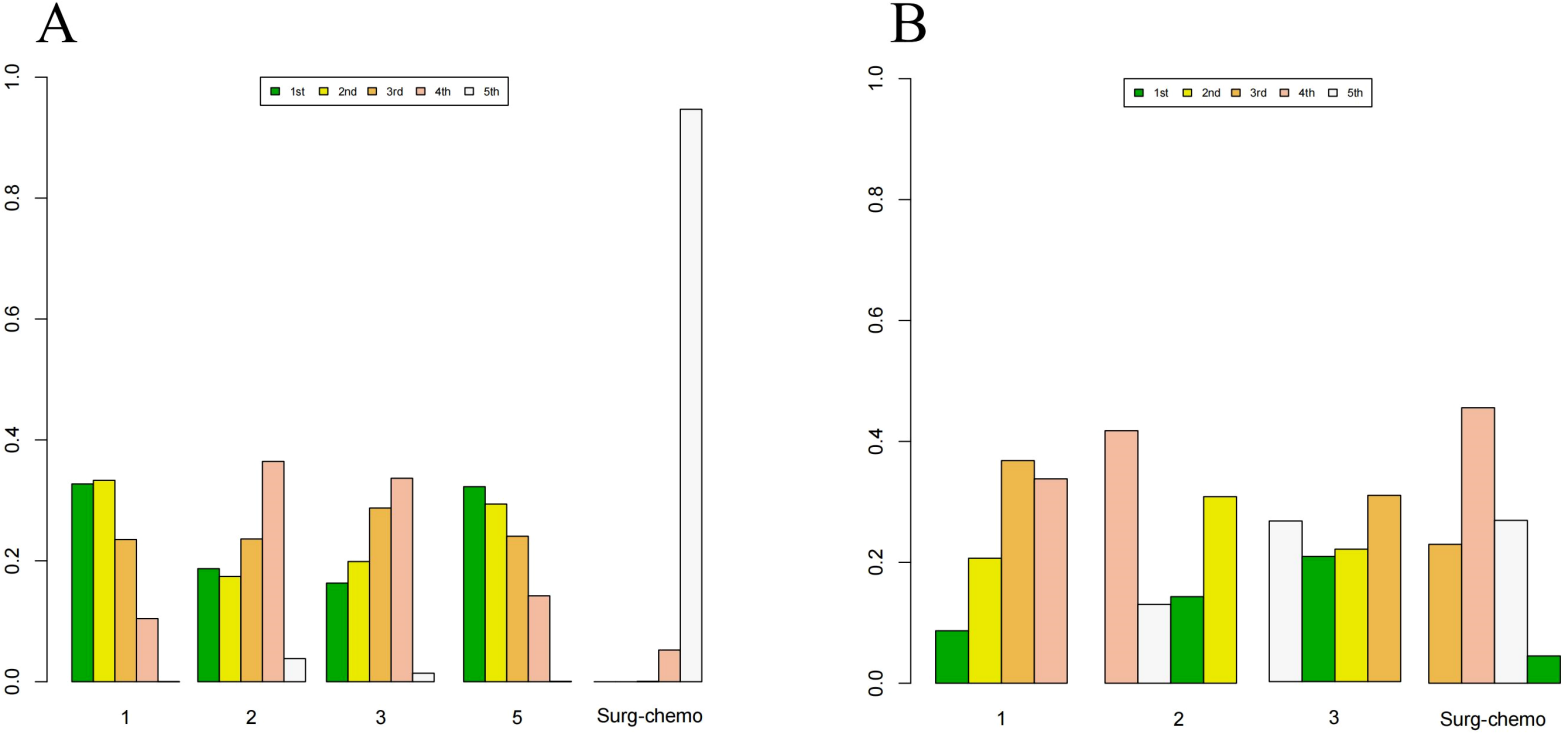
Histogram of possible ranking probabilities for each HIPEC regimen of prophylactic studies. **(A)** Ranking probability of each HIPEC regimen in OS. **(B)** Ranking probability of each HIPEC regimen in postoperative morbidity. HIPEC regimes. 1:Surg-chemo-CDDP. 2:Surg-chemo-MMC. 3:Surg-chemo-CDDP-FU. 4:Surg-chemo-CDDP-MMC. 5:Surg-chemo-OHP- 5FU.

Based on Bayesian ranking analysis (**Figures 7**, **8**; **Supplementary Tables 3–8**), for overall analysis, the top three regimens for OS are as follows: the cisplatin regimen is most likely to rank first (34.74%), followed by the oxaliplatin plus 5-fluorouracil regimen in second place (27.45%), and the mitomycin C regimen in third place (28.61%). For overall disease recurrence (**Supplementary Table 4**), the oxaliplatin plus 5-fluorouracil regimen is most likely to rank first (34.24%), the cisplatin regimen ranks second (37.52%), and the mitomycin C regimen ranks third (21.08%). Regarding peritoneal recurrence (**Supplementary Table 5**), the mitomycin C regimen is most likely to rank first (83.80%), with the cisplatin regimen ranking second (83.11%). In terms of postoperative morbidity (**Supplementary Table 6**), the mitomycin C regimen ranks first (39.31%), while the cisplatin program is second to last (23.46%).

In the subgroup analysis of prophylactic studies, the top three regimens for OS (**Supplementary Table 7**) were as follows: the oxaliplatin plus 5-fluorouracil regimen had the highest likelihood of ranking first (32.26%), followed by the cisplatin regimen (33.31%), and the cisplatin plus fluorouracil regimen (28.73%). For postoperative morbidity (**Supplementary Table 8**), the mitomycin C regimen ranked first (41.77%), while the cisplatin plus fluorouracil regimen ranked last (30.81%).

For overall analysis, the chemotherapy regimen ranked last in terms of Overall Survival (67.52%), overall disease recurrence (42.66%), and peritoneal recurrence (97.56%). However, it ranked second in terms of postoperative morbidity (37.82%)(**Supplementary Table 6**). In the subgroup analysis of prophylactic studies, chemotherapy regimens ranked last for overall survival (OS) (94.69%) and second for postoperative morbidity (45.56%).

#### 3.2.4 Funnel plots

**Figure 9:**
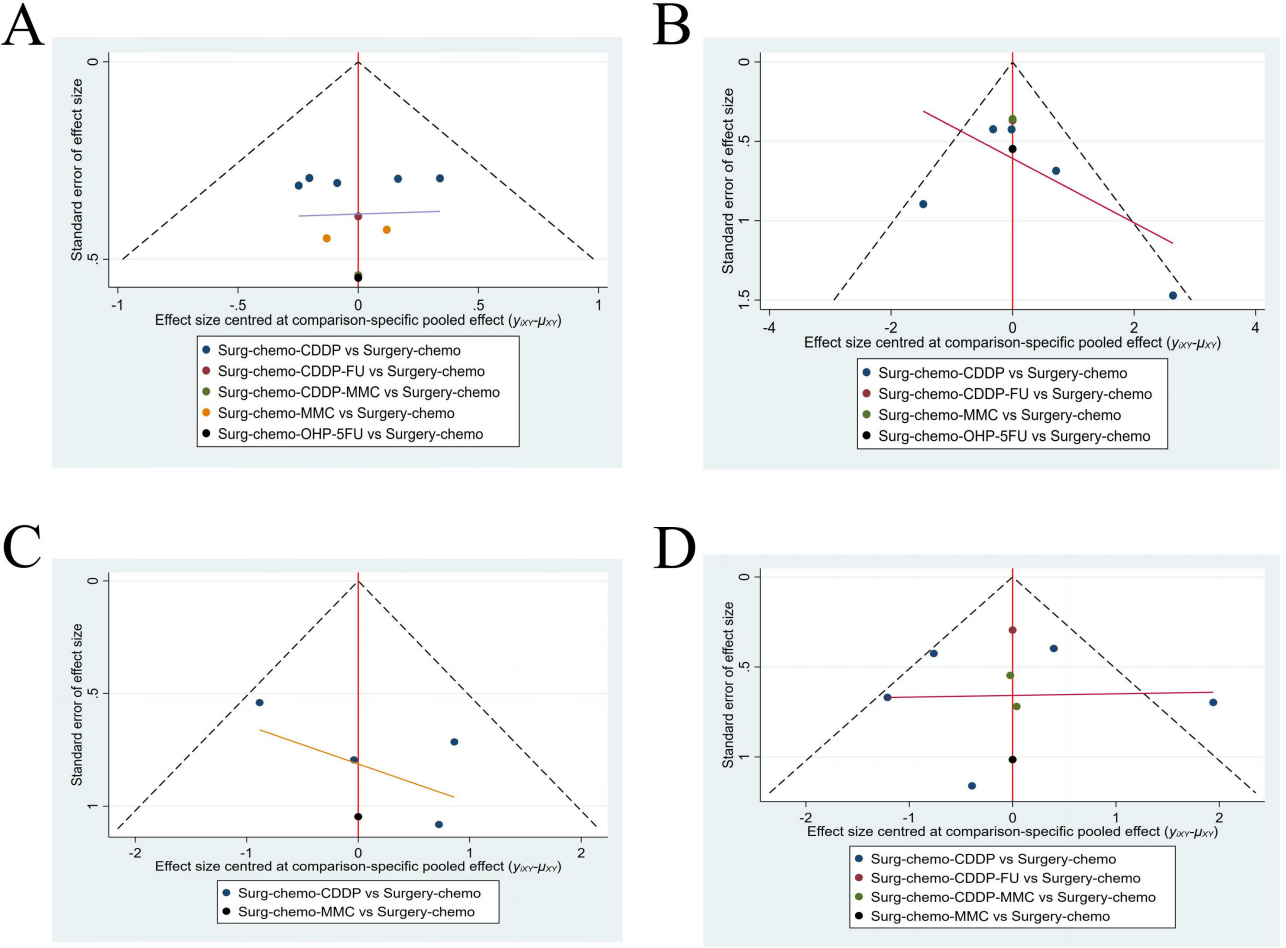
Funnel plot with HRs and ORs as an outcome indicator. **(A)** Funnel plot of OS with HR as outcome indicator. **(B)** Funnel plot of overall disease recurrence with ORs as outcome indicator. **(C)** Funnel plot of peritoneal recurrence with ORs as outcome indicator. **(D)** Funnel plot of postoperative morbidity with ORs as outcome indicator.

A funnel plot was generated to assess publication bias, using ORs and HRs as outcome indicators of effect size (**Figure 9**). The results indicated a symmetrical distribution of scatter points across the studies, with no significant outliers, suggesting a low likelihood of publication bias in this analysis.

## 4 Discussion

Despite the growing global application of HIPEC, consensus on its role in advanced gastric cancer remains elusive. Furthermore, the variation in HIPEC techniques and drug regimens has hindered the establishment of standardized clinical guidelines (31). This study synthesizes the available RCTs on HIPEC in combination with surgery and chemotherapy for advanced gastric cancer. By evaluating OS, overall disease recurrence, peritoneal recurrence, and postoperative morbidity, this study aims to identify the optimal chemotherapeutic regimen for HIPEC application.

To the best of our knowledge, this study is the first network meta-analysis of randomized controlled trials to investigate the efficacy and safety of different HIPEC regimens for the treatment of advanced gastric cancer. This study includes five HIPEC regimens, and the results indicate that most HIPEC regimens, except for the cisplatin plus mitomycin C regimen, provide a clear benefit in terms of OS. Furthermore, there were no significant differences among the HIPEC regimens, suggesting that HIPEC can deliver satisfactory survival outcomes regardless of the specific chemotherapeutic agents used. Notably, the comparable benefits observed between the cisplatin regimen and the oxaliplatin plus 5-fluorouracil regimen, as well as between the cisplatin plus fluorouracil regimen and the mitomycin C regimen, further illustrate that the differences in drug types do not impact the therapeutic effectiveness of HIPEC.

In 1988, Koga et al. (32) were the first to apply HIPEC in the treatment of gastric cancer, utilizing mitomycin C as the therapeutic agent. This drug has been extensively used in both conventional chemotherapy and HIPEC for an extended period. Mitomycin C exerts its anticancer effects by inducing cross-links between DNA strands, thereby disrupting DNA replication and transcription processes and ultimately inhibiting cancer cell division and proliferation (33).

In this study, the cisplatin regimen demonstrated the most favorable outcomes for OS, being utilized in 6 out of 11 studies, with an additional 3 studies employing it in combination. However, the cisplatin plus mitomycin C regimen did not show any significant benefit, as evidenced by its use in only two studies (19, 24). The study conducted by Rau et al. (24) indicated that the OS for both the CRS-A and CRS-HIPEC groups was 14.9 months. Among the patients included in the study, 44% had a PCI score ≥7, and 40% presented with ascites, both of which are factors associated with poor postoperative prognosis. Additionally, more than half of the patients could not undergo CRS following neoadjuvant chemotherapy. These factors collectively contributed to the study’s outcomes and impacted the OS results of the cisplatin plus mitomycin C regimen. In the subgroup analysis of this study, patients who completed CRS and achieved CCR0 showed significantly improved OS outcomes with HIPEC. This finding suggests that the completeness of CRS combined with HIPEC is the most critical factor in determining postoperative survival in patients with advanced gastric cancer, potentially laying the groundwork for future research.

Regarding tumor metastasis and recurrence, HIPEC treatment demonstrates more satisfactory effects on the peritoneum. The results of this study indicate that both the mitomycin C and cisplatin regimens provide clear benefits for peritoneal recurrence. However, none of the HIPEC regimens showed improvement in overall disease recurrence. Among these regimens, the cisplatin regimen was the most effective in terms of OS and also showed some effectiveness for peritoneal recurrence. Despite the lack of a statistically significant difference in overall disease recurrence, the cisplatin regimen still ranked first, outperforming the other regimens. This study suggests that platinum-based drugs can act on the DNA molecules of tumor cells, thereby affecting the processes of DNA replication and synthesis in tumor cells, ultimately inhibiting tumor replication (34). The disparity in the effects on overall disease recurrence versus peritoneal recurrence may be attributed to HIPEC delivering chemotherapy drugs at the peritoneal level, resulting in higher local intracellular drug concentrations. The synergistic effects of hyperthermia and chemotherapy contribute to the eradication of tumor cells within the abdominal cavity. Studies have shown that HIPEC is less effective in treating deeper subperitoneal tumor cells and extensive lymph node metastases. Meta-analyses also indicate that HIPEC does not provide a differential benefit for local tumor or lymph node recurrence and does not offer advantages in preventing liver metastasis or systemic tumor spread (35–38). However, some studies have reported advantages of HIPEC in reducing both overall and peritoneal recurrence rates in advanced gastric cancer, clearly indicating that it can improve overall recurrence outcomes (20, 39, 40). These findings suggest that the effects of HIPEC on overall metastasis and recurrence remain controversial, warranting further investigation.

Regarding safety concerns, the results of this study show no statistical difference between all HIPEC regimens and the standard treatment of surgery plus chemotherapy. Some studies have indicated that HIPEC may involve complications such as anastomotic leakage, wound infection, bowel obstruction, liver dysfunction, and significant systemic toxicity. These complications are particularly pronounced when combined with surgery, significantly increasing the incidence of grade III or higher complications, thus making perioperative management challenging (41, 42). This has led to ongoing concerns about the safety of HIPEC. However, recent meta-analyses have shown that HIPEC combined with surgery does not increase the risk of these complications. This improvement is likely due to advancements in surgical techniques, changes in drug administration methods, and more standardized HIPEC procedures (40, 43–45). In this study, the cisplatin regimen was included in five studies. Although the results across these studies did not show statistically significant differences, we are concerned that the Postoperative morbidity in the HIPEC group (7.5%) in the study by Beeharry et al. (30) was notably lower than that of the control group (15%). This disparity may have influenced the overall data; however, the absence of significant bias across the studies.

Nonetheless, perioperative mortality remains a significant concern, despite the lack of sufficient data on this issue in the studies included in this paper. Previous research has demonstrated that chemotherapy combined with HIPEC is linked to considerable systemic toxicity, particularly manifesting as respiratory and renal failure, with reported mortality rates ranging from 3% to 10% (46). This indicates that the effects are not confined to the abdominal cavity, but that the entire body undergoes substantial stress during treatment. Additionally, adjunctive treatments such as antiemetic, antiallergic, and steroid therapies during HIPEC can significantly reduce adverse reactions, enhancing patient tolerance to the treatment, and thereby improving clinical outcomes and quality of life (47).

This study also conducted a subgroup analysis of prophylactic HIPEC. Initially, HIPEC was primarily employed in cases with confirmed peritoneal metastases (11). However, as research has progressed, it has been recognized that even gastric cancer patients without overt peritoneal metastases or positive abdominal cytology are at risk of developing metachronous peritoneal carcinomatosis (PC) postoperatively. In a study involving radical D2 gastrectomy for gastric cancer, tumor recurrence was observed in approximately 50% of patients, with 15.5% developing metachronous PC (48). Currently, the standard treatment for locally advanced gastric cancer includes neoadjuvant chemotherapy followed by radical gastrectomy with D2 lymphadenectomy, and adjuvant chemotherapy. Nevertheless, due to the presence of peritoneal micrometastases, peritoneal recurrence still occurs in about 45% of patients after surgery (49, 50).The primary concern regarding prophylactic HIPEC is whether it can improve patient prognosis while simultaneously controlling postoperative complications. A clinical study involving HIPEC in patients with serosal invasion gastric cancer demonstrated that HIPEC could enhance prognosis with acceptable postoperative morbidity (51).

This meta-analysis performed a subgroup analysis focusing on OS and postoperative morbidity to assess the impact of prophylactic HIPEC in gastric cancer patients. Notably, while the overall analysis showed a survival benefit from the mitomycin C regimen, this benefit was not observed in the prophylactic subgroup (HRs = 0.61, 95% CI: 0.35–1.06). We hypothesize that this may be due to the fact that only one study in the prophylactic subgroup utilized the mitomycin C regimen, which given the small sample size, may have contributed to the variability of the results. Other HIPEC regimens in the subgroup analysis produced similar outcomes to the overall analysis. Regarding postoperative morbidity, the findings indicated no significant differences between HIPEC regimens and standard chemotherapy, which was consistent with the overall analysis. These results suggest that HIPEC provides a substantial preventive effect against peritoneal metastases in locally advanced gastric cancer, offering a notable survival benefit without significantly increasing complication rates, making it a promising approach for broader clinical application.

Despite the numerous findings obtained in this study, several limitations exist. Firstly, our inclusion criteria were restricted to RCT studies involving surgery combined with HIPEC and chemotherapy, resulting in the inclusion of only 11 studies. Although we conducted a thorough search, the number of studies remains insufficient. Secondly, the majority of these studies were conducted in Asian countries, predominantly involving Asian populations, which may limit the generalizability of our conclusions to other racial groups. Additionally, we did not provide detailed dosing regimens for each chemotherapy protocol, and the duration of HIPEC varied across studies, which may influence treatment outcomes. Although a general consensus exists, these variations could still impact the results (52). Thirdly, the differences in the specific chemotherapy regimens employed could affect the study results. Lastly, due to the limited number of studies, the cisplatin plus fluorouracil regimen and the oxaliplatin plus 5-fluorouracil regimen were each only represented by one study, which may introduce bias. Moreover, the research on peritoneal recurrence involved only two HIPEC regimens, which is not comprehensive.

Our study results indicate that, compared to chemotherapy combined with surgery, HIPEC treatment offers significant benefits in OS and peritoneal recurrence, with no notable differences in postoperative morbidity. Regarding HIPEC drug regimens, although no single regimen demonstrated clear benefits across all outcomes, the cisplatin regimen showed promising results in various endpoints, warranting further research and application.

## Data Availability

The original contributions presented in the study are included in the article/**Supplementary Material**. Further inquiries can be directed to the corresponding author.
